# Nonclinical study and applicability of the absorbed dose conversion method with a single biodistribution measurement for targeted alpha-nuclide therapy

**DOI:** 10.1186/s40658-021-00425-z

**Published:** 2021-12-11

**Authors:** Tetsuya Sakashita, Shojiro Matsumoto, Shigeki Watanabe, Hirofumi Hanaoka, Yasuhiro Ohshima, Yoko Ikoma, Naoyuki Ukon, Ichiro Sasaki, Tatsuya Higashi, Tetsuya Higuchi, Yoshito Tsushima, Noriko S. Ishioka

**Affiliations:** 1 Quantum Beam Science Research Directorate, National Institutes for Quantum Science and Technology, 1233 Watanuki-machi, Takasaki, 370-1292 Japan; 2 Department of Bioimaging Information Analysis, Gunma University Graduate School of Medicine, 3-39-22 Showa, Maebashi, 371-8511 Japan; 3 Department of Molecular Imaging and Theranostics, National Institutes for Quantum Science and Technology, 4-9-1 Anagawa, Inage-ku, Chiba, 263-8555 Japan; 4 Advanced Clinical Research Center, Fukushima Medical University, 1 Hikariga-oka, Fukushima, 960-1295 Japan; 5 Department of Diagnostic Radiology and Nuclear Medicine, Gunma University Graduate School of Medicine, 3-39-22 Showa, Maebashi, 371-8511 Japan

**Keywords:** Targeted alpha-nuclide therapy, Dose conversion, Biodistribution, Pharmacokinetics, RAP

## Abstract

**Background:**

We recently reported a new absorbed dose conversion method, RAP (**RA**tio of **P**harmacokinetics), for ^211^At-*meta*-astatobenzylguanidine (^211^At-MABG) using a single biodistribution measurement, the percent injected dose/g. However, there were some mathematical ambiguities in determining the optimal timing of a single measurement of the percent injected dose/g. Thus, we aimed to mathematically reconstruct the RAP method and to examine the optimal timing of a single measurement.

**Methods:**

We derived a new formalism of the RAP dose conversion method at time *t*. In addition, we acquired a formula to determine the optimal timing of a single measurement of the percent injected dose/g, assuming the one-compartment model for biological clearance.

**Results:**

We investigated the new formalism’s performance using a representative RAP coefficient with radioactive decay weighting. Dose conversions by representative RAP coefficients predicted the true [^211^At]MABG absorbed doses with an error of 10% or less. The inverses of the representative RAP coefficients plotted at 4 h post-injection, which was the optimal timing reported in the previous work, were very close to the new inverses of the RAP coefficients 4 h post-injection. Next, the behavior of the optimal timing was analyzed by radiolabeled compounds with physical half-lives of 7.2 h and 10 d on various biological clearance half-lives. Behavior maps of optimal timing showed a tendency to converge to a constant value as the biological clearance half-life of a target increased. The areas of optimal timing for both compounds within a 5% or 10% prediction error were distributed around the optimal timing when the biological clearance half-life of a target was equal to that of the reference. Finally, an example of RAP dose conversion was demonstrated for [^211^At]MABG.

**Conclusions:**

The RAP dose conversion method renovated by the new formalism was able to estimate the [^211^At]MABG absorbed dose using a similar pharmacokinetics, such as [^131^I]MIBG. The present formalism revealed optimizing imaging time points on absorbed dose conversion between two radiopharmaceuticals. Further analysis and clinical data will be needed to elucidate the validity of a behavior map of the optimal timing of a single measurement for targeted alpha-nuclide therapy.

**Supplementary Information:**

The online version contains supplementary material available at 10.1186/s40658-021-00425-z.

## Background

Pheochromocytoma includes cases of systemic metastasis, which occurs in about 10–15% of patients and progresses into malignant pheochromocytoma [1]. *Meta*-[^131^I] iodobenzylguanidine (^131^I-MIBG) is a radiopharmaceutical for the systemic treatment of patients with metastatic pheochromocytoma. ^131^I-MIBG, an analog of guanethidine, accumulates in adrenergic tissue by the same mechanism as that of norepinephrine through the norepinephrine transporter (NET) [2]. Treatment with ^131^I-MIBG has shown limited efficacy even when administered at high radioactivity, such as more than 7.4 GBq [1]. Thus, new effective approaches are needed for patients with metastatic pheochromocytoma. *Meta*-[^211^At]astatobenzylguanidine (^211^At-MABG), an alpha-emitting radiopharmaceutical, might be an alternative to ^131^I-MIBG for the treatment of malignant pheochromocytoma, because the uptake mechanisms of these radiopharmaceuticals are similar. Alpha particles have high LET and very short ranges in tissues compared with electrons. Therefore, ^211^At-MABG should be more effective than ^131^I-MIBG.

Dosimetry for therapy with alpha-particle emitters is currently in a challenging stage. Several studies based on planar images of ^211^At obtained using gamma cameras have been reported [3, 4]. However, there are still issues with the spatial resolution and quantification of the images because of the lack of attenuation correction. Ukon et al. [5] assessed absorbed doses in humans by measuring and extrapolating the in vivo distribution in mice. In ^131^I-MIBG therapy, diagnostic imaging by ^131^I-scintigraphy and/or SPECT during ^131^I-MIBG treatment would be performed occasionally as part of follow-up care, because such imaging is of clinical prognostic value. Taken together, predicting the organ or tumor tissue absorbed dose of such patients in ^211^At-MABG treatment using ^131^I-MIBG image data would be useful for planning ^211^At-MABG therapy. Therefore, it is important to develop methods for estimating ^211^At-MABG doses using ^131^I-MIBG biodistribution data.

At present, one of the issues in systemic radiopharmaceutical therapy or TAT is the optimization of imaging time points [6]. The previous work of Madsen et al. [6] revealed a single time to estimate the total integrated activity and absorbed dose within 10% accuracy. However, their method only works if the biological half-life is well known. That is, we cannot apply their method if it is unknown. Recently, we reported a novel dose conversion method, RAP **(RA**tio of **P**harmacokinetics), using the percent injected dose/g (%ID/g) [7]. In that study, we extended optimizing imaging time points on the absorbed dose conversion between two radiopharmaceuticals and demonstrated that the RAP dose conversion method could estimate ^211^At-MABG absorbed doses from a single measurement of %ID/g and the pharmacokinetics of ^131^I-MIBG. However, this was still an insufficient mathematical approach for the timing of a single measurement of %ID/g. Therefore, we aimed to describe the RAP method mathematically without proportional relations and to examine the timing of a single measurement of %ID/g.

## Materials and methods

### Simulation datasets

On time series of biodistribution profiles in our previous Monte Carlo simulation work on ^211^At-MABG, ^131^I-MIBG, ^77^Br-MBBG, and ^125^I-MIBG, we used a total of 8,000 simulation (virtual experiment) datasets [7]. In the present study, we applied a median of 200 simulation datasets at each organ or tumor tissue. The simulation was carried out based on the biodistribution profiles of three reports at several time points of seven organs (heart, liver, kidney, intestine, blood, adrenals, stomach) and tumor tissue [8–10]. One of these reports, by Vaidyanathan et al., reported the biodistributions of ^211^At-MABG and ^131^I-MIBG in nude mice with SK-N-SH human neuroblastoma xenografts [8]. In the second, by Ohshima et al. [9], a rat PC12 pheochromocytoma model was used to examine the antitumor effects of ^211^At-MABG. The third report, by Watanabe et al. [10], analyzed the biodistributions of ^77^Br-MBBG and ^125^I-MIBG using PC12 xenografts. We have labeled the simulated biodistribution datasets created from these previous reports as [^211^At]MABG [8], [^131^I]MIBG [8], [^211^At]MABG [9], [^77^Br]MBBG [10], and [^125^I]MIBG [10], respectively.

### New formalism of RAP method at time *t*

In our previous work, we could not present a sufficient mathematical approach to the timing of a single measurement. The mathematical basis of the RAP method was the proportional relation, which was derived from our findings of the good correlation between the absorbed dose ratio and the %ID/g ratio [7] as follows:

1 $$D \left({}^{211}At\right)\propto D \left({}^{131}I\to {}^{211}At\right)\times \frac{1}{\frac{{(\mathrm{\%ID}/\mathrm{g})}_{{}^{131}I}}{{(\mathrm{\%ID}/\mathrm{g})}_{{}^{211}At}}}$$

where *D* (^211^*At*) is the absorbed dose of ^211^At, *D* (^131^*I* → ^211^*At*) is the absorbed dose conversion using the exchange of the physical half-life (HL) in the activity concentration, and $\frac{1}{\frac{{(\mathrm{\%ID}/\mathrm{g})}_{{}^{131}I}}{{(\mathrm{\%ID}/\mathrm{g})}_{{}^{211}At}}}$ is the RAP coefficient defined in a previous work [7]. There were some mathematical ambiguities.

Here, we show a new derivation method for the RAP formula based on the activity concentration (kBq/g) of an organ or tumor tissue, *C* (*t*). In the previous work, *C* (*t*) was expressed using the two-biological-compartment model for normal organs except adrenals:

2 $$C\left(t\right)={C}_{0}exp\left(-\frac{ln(2)}{{T}_{p}}t\right)\left\{fexp(-\frac{ln(2)}{{T}_{b1}}t)+(1-f)exp(-\frac{ln(2)}{{T}_{b2}}t)\right\},$$

where *C*(*t*) is the activity concentration for the normal organ at time (s) *t* post-injection, *C*_*0*_ is the initial activity concentration, *T*_*p*_ is the physical HL time (s), and *f* and (1 − *f*) are the fractions of the two biological compartments on clearance. *T*_*b1*_ and *T*_*b2*_ are the corresponding HL times (s) for fast and slow biological clearances, respectively. Or, in the case of adrenals and tumor tissue, the following one-compartment equation was used:

3 $$C\left(t\right)={C}_{0}\left(1-\mathrm{exp}\left(-\frac{\mathrm{ln}\left(2\right)}{{T}_{up}}t\right)\right)exp\left(-\frac{\mathrm{ln}\left(2\right)}{{T}_{p}}t\right) exp(-\frac{ln(2)}{{T}_{b1}}t),$$

where *T*_*up*_ is the HL time of uptake (s).

In this study, we re-expressed activity concentration Eqs. (2) and (3) as follows using injected activity concentration *IAC*_0_ (kBq/g) and %ID/g (*t*) at time (s) *t* post-injection:

4 $$C\left(t\right)={IAC}_{0}exp(-\frac{ln(2)}{{T}_{p}}t) ({\mathrm{\%ID}/\mathrm{g})}\left(t\right)$$

where in the previous simulation work, we assumed an injection with 100 kBq of ^211^At-MABG in 100 μL of PBS into a tail vein and around 1 MBq as the total activity of a mouse. In our calculation, we also assumed that 1 ml of PBS is equal to 1 g. Next, we applied this equation to two radiolabeled compounds, *A*_1_ and *A*_2_.

4-1 $${C}_{{A}_{1}\left(t\right)}={IAC}_{0}{{}_{A}}_{1}exp\left(-\frac{\mathrm{ln}\left(2\right)}{{T}_{p{A}_{1}}}t\right) ({\mathrm{\%ID}/\mathrm{g})}_{{A}_{1}}\left(t\right) \mathrm{ and}$$

4-2 $${C}_{{A}_{2}\left(t\right)}={IAC}_{0}{{}_{A}}_{2}exp\left(-\frac{\mathrm{ln}\left(2\right)}{{T}_{p{A}_{2}}}t\right) ({\mathrm{\%ID}/\mathrm{g})}_{{A}_{2}}\left(t\right).$$

By dividing and transforming both sides of the (4–1) and (4–2) equations, we described the following new derivative relation for the RAP formalism:

5 $${C}_{{A}_{1}\left(t\right)}={C}_{{A}_{2}\left(t\right)}{\frac{{IAC}_{0}{{}_{A}}_{1}}{{IAC}_{0}{{}_{A}}_{2}}\frac{\mathrm{exp}\left(-\frac{\mathrm{ln}\left(2\right)}{{T}_{p{A}_{1}}}t\right)}{\mathrm{exp}\left(-\frac{\mathrm{ln}\left(2\right)}{{T}_{p{A}_{2}}}t\right)}\frac{1}{\frac{({\mathrm{\%ID}/\mathrm{g})}_{{A}_{2}}\left(t\right)}{({\mathrm{\%ID}/\mathrm{g})}_{{A}_{1}}\left(t\right)}}}.$$

Here, $\frac{1}{\frac{({\mathrm{\%ID}/\mathrm{g})}_{{A}_{2}}\left(t\right)}{({\mathrm{\%ID}/\mathrm{g})}_{{A}_{1}}\left(t\right)}}$ is the RAP coefficient at *t*. We assumed *IAC*_0_ to 1 MBq/ml for both radiolabeled compounds *A*_1_ and *A*_2_ and set *IAC*_0__*A*_1_/*IAC*_0__*A*_2_ to 1. If the injected activity concentration is x MBq/ml, we should multiply the calculated result by x times. To calculate a time integration activity concentration (TIAC) (kBq-h/g) of radiolabeled compounds *A*_1_ and *A*_2_, we numerically integrated Eq. (5) with a 1-h interval.

The absorbed radiation dose (Gy), *D*, for normal or tumor tissue was calculated according to the following modified MIRD formalism:

6 $$D=1000\cdot TIAC \cdot E\cdot F\cdot P$$

where the first factor, 1000, is for converting the TIAC (kBq-h/g) from kBq to Bq. The energy emitted by ^211^At, *E*, is assumed to be solely from the alpha disintegrations, corresponding to 6.9 MeV/Bq-s [11]. The absorbed fraction, *F*, is set to 1, since it is assumed that all energy emitted by ^211^At is absorbed by the source tissue or organ. *P* is the coefficient for converting from g to kg, 1000. Finally, the absorbed dose (J/kg = Gy) was calculated using the relation of 1.602 10^–13^ (J/MeV).

### Framework for practical use of the RAP dose conversion

In the present work, we set radiolabeled compound A_2_, which has a well-known biological kinetics, as a reference, and radiolabeled compound A_1_ as a target with unknown biological kinetics. Our goal is to convert from the absorbed dose of A_2_ to that of A_1_. Integrating Eq. (5) leads to a TIAC (kBq-h/g) of A_1_, but, in the case of unknown biological kinetics of A_1_, integration would be difficult. On the other hand, the physical part of Eq. (5), that is, Eq. (5) except for the RAP coefficient, could be easily integrated if the pharmacokinetics of A_2_ is well known. Here, it should be noted that a single measurement of %ID/g has been used to demonstrate successful RAP dose conversion [7]. In short, we needed to work on simplifying the integration of the RAP coefficients and used simulation datasets from the previous work to achieve that.

In the first attempt, the analysis of RAP coefficients, we numerically integrated the physical part of Eq. (5) except for the RAP coefficient. We also calculated the mean value of the target’s radioactive decay weighted RAP coefficients during the evaluation period, representative RAP coefficient. TIACs were obtained by multiplying the integral value of the physical part by the representative RAP coefficient. Finally, absorbed doses were estimated using Eq. (6). We labeled the absorbed dose based on the TIACs that were considered the physical part of the integration as “with HL,” and that considered TIACs multiplied by the representative RAP coefficient as “with HL + RAP.” The evaluated absorbed doses in previous methods, e.g., Sato’s work [12], using the distribution of therapeutic pharmaceuticals estimated from the images of companion diagnostics, correspond to our present results for “with HL.” The previous method could not sufficiently consider the biological clearance of the therapeutic pharmaceuticals themselves. Thus, we compared “with HL” and “with HL + RAP.”

In the next attempt, the generalization of the RAP method, we acquired the formula for the optimal timing of a single measurement of %ID/g. Here, the exponential formulas of two or more terms cannot be combined into one term with respect to time in Eq. (3), and we could not obtain the optimal timing analytically using this equation. Thus, we adopted a compartment model without the uptake phase and obtained the optimal timing, *Opt_t*. In addition, our previous findings suggested that a ratio of exponential time integrals for the two models could be expressed by using the ratio of exponential values at a time *t* for them [7]. That is, using the RAP coefficient and TIACs, the RAP coefficient can be expressed as follows:

7 $${\text{RAP coefficient}} = { }\frac{{TIAC\_A_{1} }}{{TIAC\_A_{2} with HL}}.$$

Here, to analytically solve the optimal timing at which the RAP coefficient satisfies Eq. (7), we assume the one-compartment model for biological clearance. Then, the optimal timing, *Opt_t,* is obtained as follows (Additional file 1):

8 $$Opt\_t = \frac{1}{{ - \ln 2 \left( {\frac{1}{{T_{{bA_{1} }} }} - \frac{1}{{T_{{bA_{2} }} }}} \right)}}\ln \frac{{\frac{1}{{\frac{1}{{T_{{pA_{1} }} }} + \frac{1}{{T_{{bA_{1} }} }}}}\left[ {{\text{exp}}\left( { - \ln 2\left( {\frac{1}{{T_{{pA_{1} }} }} + \frac{1}{{T_{{bA_{1} }} }}} \right)t} \right)} \right]_{{t_{0} }}^{{t_{1} }} }}{{\frac{1}{{\frac{1}{{T_{{pA_{1} }} }} + \frac{1}{{T_{{bA_{2} }} }}}}\left[ {{\text{exp}}\left( { - \ln 2\left( {\frac{1}{{T_{{pA_{1} }} }} + \frac{1}{{T_{{bA_{2} }} }}} \right)t} \right)} \right]_{{t_{0} }}^{{t_{1} }} }},$$

where *t*_0_ and *t*_1_ are the start and end times of the evaluation period.

Finally, we present an example of dose conversion by the RAP method using a mathematical formula for the optimal timing, Eq. (8), for a single measurement of %ID/g.

## Results

### Analysis of the RAP coefficients

**Fig. 1:**
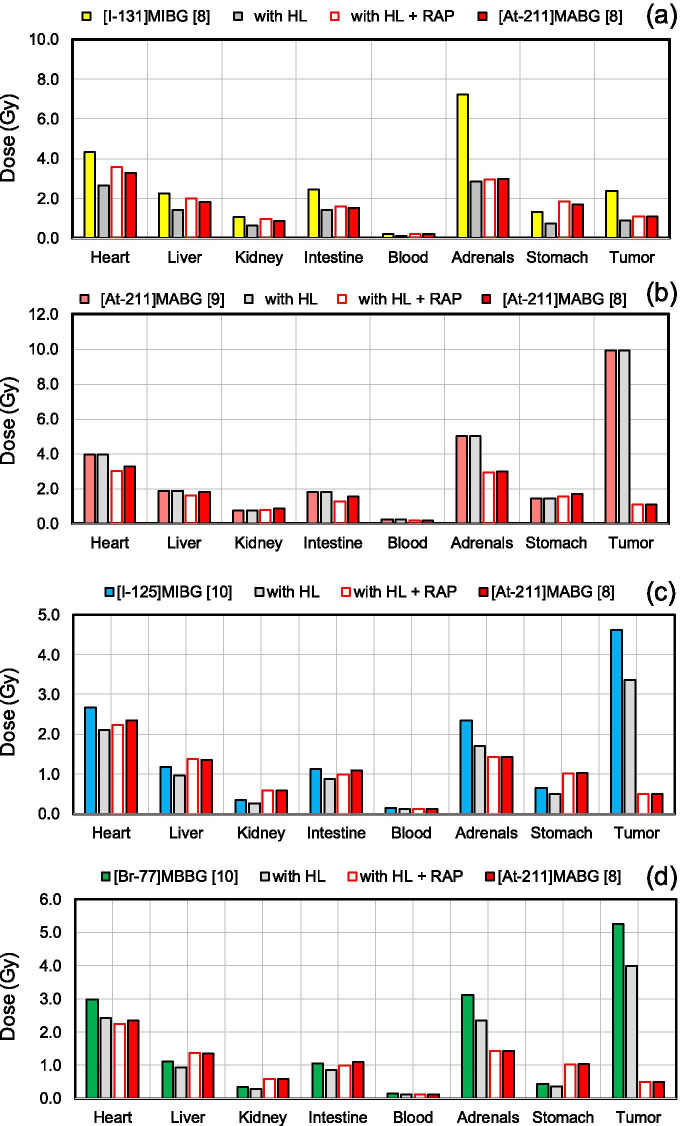
Comparison between converted absorbed doses of [^131^I]MIBG [8], [^211^At]MABG [9], [^125^I]MIBG [10], and [^77^Br]MBBG [10] and the true target absorbed dose of [^211^At]MABG [8]. **a** Converted absorbed doses from [^131^I]MIBG [8] as a reference, and absorbed dose of [^211^At]MABG [8] as a true target. [^131^I]MIBG [8] indicates the absorbed dose of an organ or tumor tissue when alpha rays derived from ^211^At were emitted by the number of radioactive decays of ^131^I. “with half-life (HL)” shows a converted absorbed dose of [^131^I]MIBG [8] corrected by the physical half-life of ^211^At, indicating the physical part of Eq. (5).“with HL + RAtio of Pharmacokinetics (RAP)” is an absorbed dose of “with HL” converted using the representative RAP coefficient. **b** Same as (**a**) except that [^211^At] MABG [9] is a reference. **c** Same as (**a**) except that [^125^I]MIBG [10] is a reference. **d** Same as (**a**) except that [^77^Br]MBBG [10] is a reference

Figure 1 shows the converted absorbed doses of the references, the “with HL”-corrected ones, the “with HL + RAP” ones, and the true absorbed dose of the [^211^At]MABG [8] target. Most “with HL + RAP” converted absorbed doses were closer to the true absorbed dose of [^211^At]MABG than the “with HL” ones. Average percent differences between converted and true absorbed [^211^At]MABG [8] doses were − 23% in “with HL” of [^131^I]MIBG [8] and 7% in “with HL + RAP.” Similarly, those of [^211^At]MABG [9] were 112% and − 7%, those of [^125^I]MIBG [10] were 53% and − 2%, and those of [^77^Br]MBBG [10] were 74% and − 2% (Table 1). These results showed that the dose conversions by the representative RAP coefficients were able to predict the true value with an error of 10% or less.

**Fig. 2:**
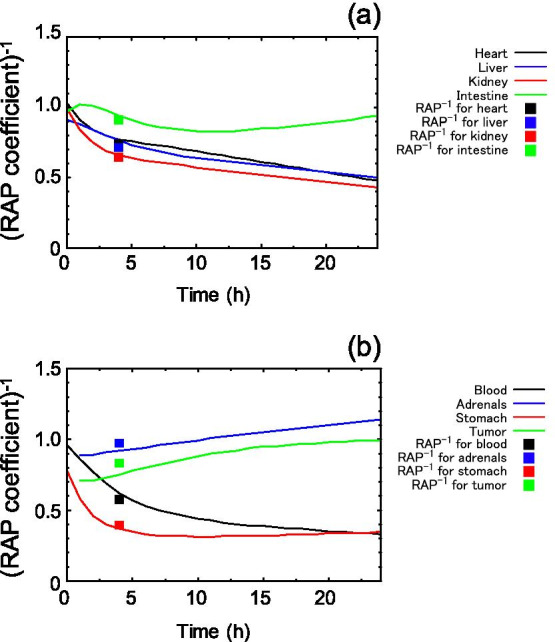
Inverse of the RAP coefficient at *t* (solid line) and the representative RAP coefficient (square) on [^131^I]MIBG [8]. **a** Heart, liver, kidney, and intestine. **b** Blood, adrenals and stomach, and tumor tissue

To examine the ratio in %ID/g of a reference to a target, we plotted the inverse of the RAP coefficient (Fig. 2). The inverses of the RAP coefficients at *t* changed over time in the seven organs and the tumor tissue. The inverses of the representative RAP coefficients were plotted at 4 h post-injection, which was the optimal timing reported in the previous work [7]. As a result, we found that the inverse values of RAP coefficients 4 h post-injection and the representative RAP coefficient were very close to each other. Taken together, these results suggest that the new RAP coefficients at 4 h post-injection corresponded to the RAP coefficients in the previous work.

### Behavior of optimal timing of a single biodistribution measurement

**Fig. 3:**
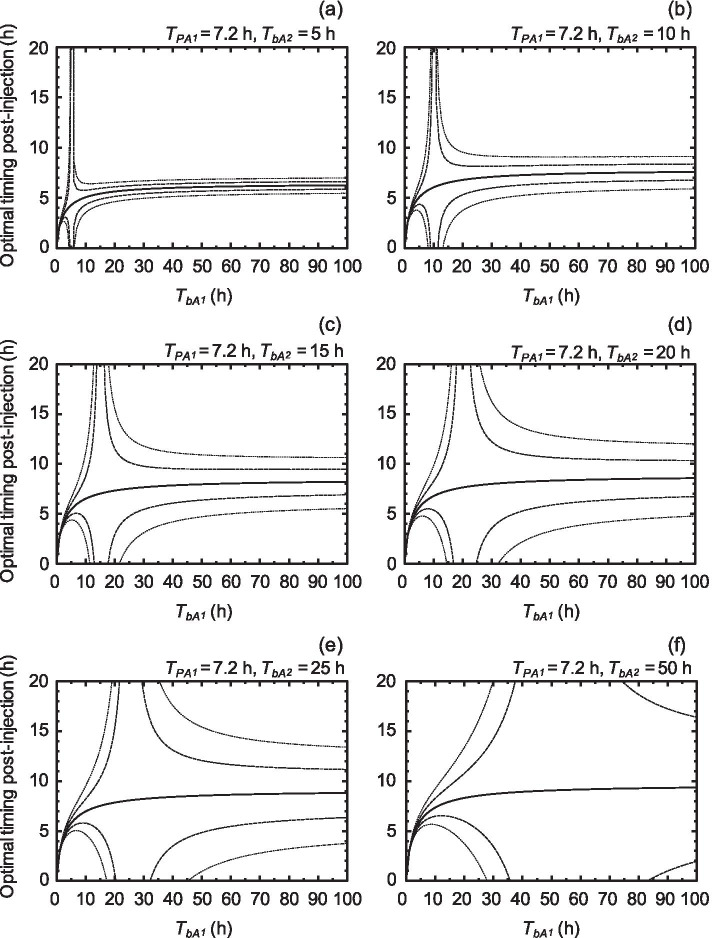
Behavior maps of an optimal timing (solid line) on ^211^At-labeled target compounds. *T*_*pA1*_ and *T*_*bA*1_ are the physical HL and HL of the biological clearance of a target, respectively. *T*_*bA*2_ is the half-life of the biological clearance of a reference. Behavior maps of *T*_*bA*2_ in these cases of 5, 10, 15, 20, 25, and 50 h are presented in the panels of (**a**), (**b**), (**c**), (**d**), (**e**), and (**f**), respectively. The dashed and dotted lines represent optimal timing in the cases of 0.95 and 1.05, and 0.9 and 1.1 times converted absorbed doses, respectively

The behavior of an optimal timing on ^211^At-labeled A_1_ was estimated depending on the HLs of the biological clearance of a target, *T*_*bA*1_, from 0 to 100 h and the HLs of the biological clearance of a reference, *T*_*bA*2_, of 5, 10, 15, 20, 25, and 50 h (Fig. 3). Here, we set the end time of the evaluation period to 72 h, which was 10 times the HL of ^211^At. In addition, we plotted 0.9, 0.95, 1.05, and 1.1 times TIACs of A_1_ in order to understand the area of optimal timing where the absorbed dose could be estimated within a 5% or 10% prediction error. As shown in Fig. 3, the optimal timing tended to converge to a constant value as *T*_*bA*1_ increased. Interestingly, all optimal timing values were acceptable when *T*_*bA*1_ was equal to *T*_*bA*2_. The area of optimal timing within a 5% or 10% prediction error on average was distributed around the intersection where the curve of optimal timing agreed with the line where *T*_*bA*1_ was equal to *T*_*bA*2_.

**Fig. 4:**
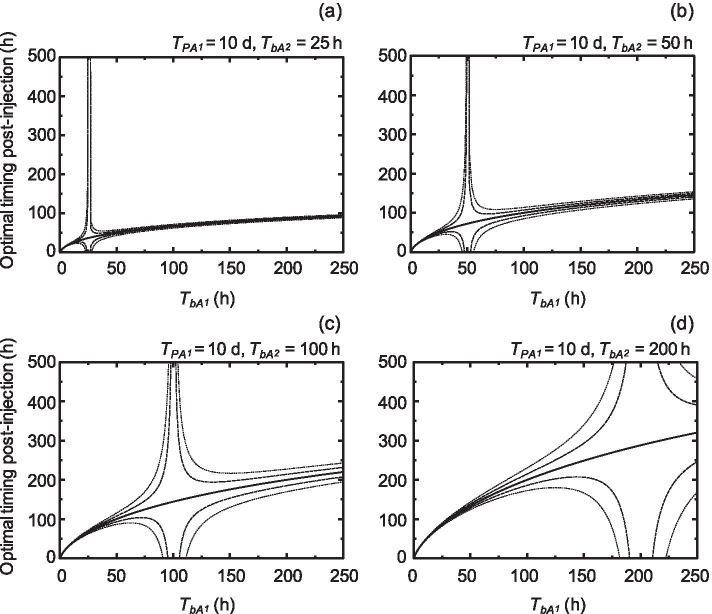
Behavior maps of optimal timing (solid line) on radiolabeled target compounds with a physical HL of 10 d. *T*_*pA1*_ and *T*_*bA*1_ are the physical HL and HL of the biological clearance of a target, respectively. *T*_*bA*2_ is the half-life of the biological clearance of a reference. Behavior maps of *T*_*bA*2_ in these cases of 25, 50, 100, and 200 h are presented in the panels of (**a**), (**b**), (**c**), and (**d**), respectively. The dashed and dotted lines represent optimal timing in the cases of 0.95 and 1.05, and 0.9 and 1.1 times converted absorbed doses, respectively

**Fig. 5:**
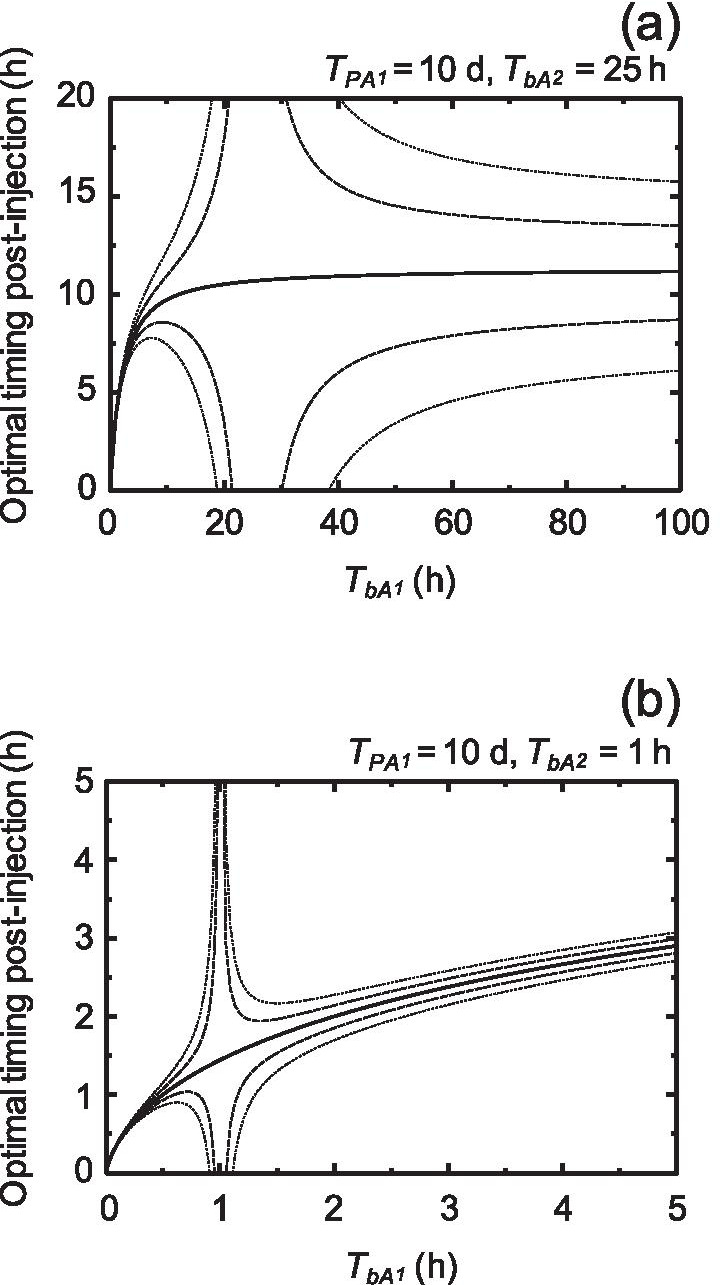
Behavior maps of an optimal timing (solid line) on radiolabeled target compounds with a physical HL of 10 d. *T*_*pA1*_ and *T*_*bA*1_ are the physical HL and HL of the biological clearance of a target, respectively. **a** HL of the biological clearance of a reference, *T*_*bA*2_, of 25 h. **b** HL of the biological clearance of a reference, *T*_*bA*2_, of 1 h. The dashed and dotted lines represent optimal timing in the cases of 0.95 and 1.05, and 0.9 and 1.1 times converted absorbed doses, respectively

Figure 4 shows the behavior of an optimal timing on A_1_ with a physical HL of 10 d, e.g., ^225^Ac, depending on *T*_*bA*1_ from 0 to 250 h and *T*_*bA*2_ of 25, 50, 100, and 200 h. Here, we set the end time of the evaluation period to 2,400 h. The optimal timing also tended to converge to a constant value as *T*_*bA*1_ increased, and the convergence value was larger than that of ^211^At. The area of optimal timing within a 5% or 10% prediction error was also distributed around the intersection where the curve of optimal timing agreed with the line where *T*_*bA*1_ was equal to *T*_*bA*2_. In addition, an enlarged view of the behavior map at 25 h *T*_*bA*2_ shows that the behavior was almost the same as that for ^211^At except for a different convergence value (Fig. 5a). Moreover, short HLs of biological clearance in *T*_*bA*1_ and *T*_*bA*2_ displayed short optimal timing (Fig. 5b). These results suggest that the area of optimal timing depended on both *T*_*bA*1_ and *T*_*bA*2_ and that the convergence value of the optimal timing was controlled by the physical HL time of radiolabeled compound A_1_.

### Example of RAP dose conversion using optimal timing of a single biodistribution measurement

Analysis of RAP coefficients and of a behavior map of the optimal timing of a single measurement of %ID/g confirmed the practical application of the RAP method. Here, we present a case of the heart in [^131^I]MIBG [8] as a reference and [^211^At]MABG [8] as a target, using the simulation datasets from the previous work [7].

**Fig. 6:**
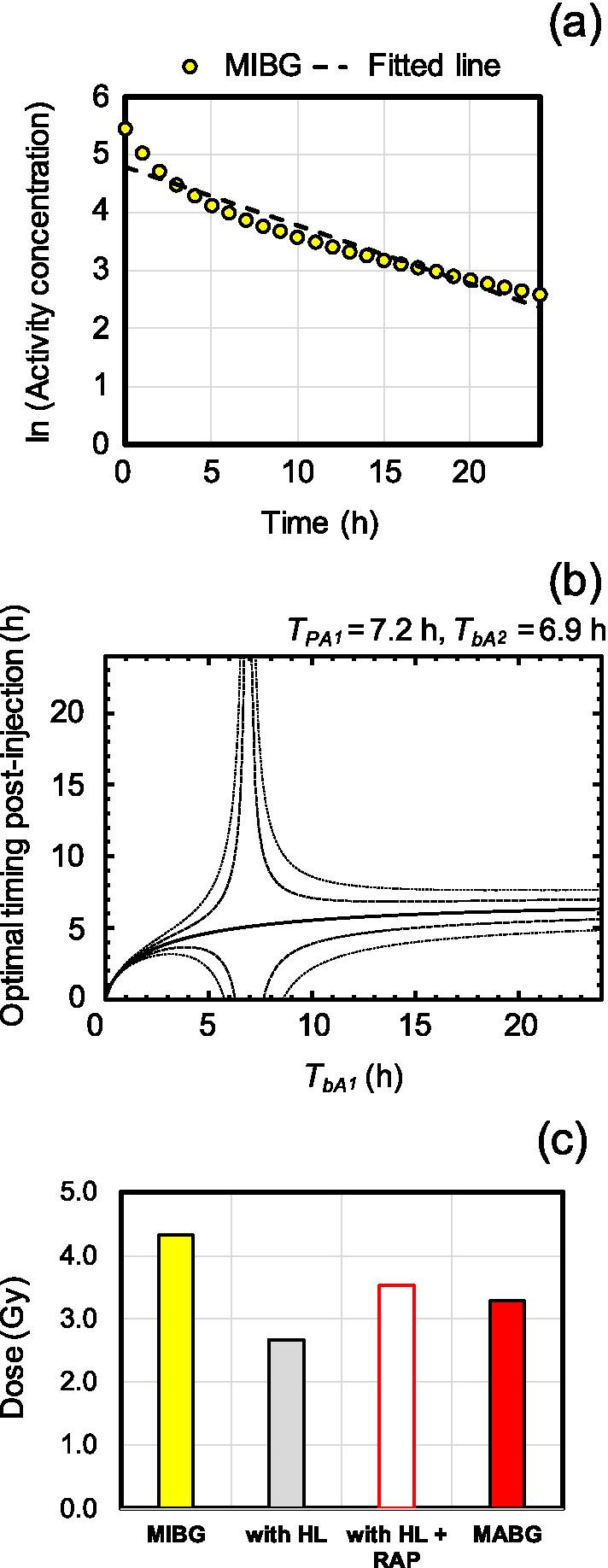
Sample of a RAP dose conversion using an optimal timing behavior map for a single biodistribution measurement (%ID/g) on ^211^At-labeled target compounds. First, **a** plotted values converted to logarithms of [^131^I]MIBG [8] corrected by the physical half-life of ^131^I, i.e., a biological component, and fitted by the linear function (the dashed line). Second, **b** draws the behavior map of an optimal timing (solid line) on a target compound with a physical HL of 7.2 h and a reference with a 6.9 h HL of biological clearance, *T*_*bA*2_, which was derived from the fitting curve on panel (**a**). The dashed and dotted lines represent optimal timing in the cases of 0.95 and 1.05, and 0.9 and 1.1 times converted absorbed doses, respectively. Finally, **c** converted absorbed doses of [^131^I]MIBG [8], “with HL,” “with HL + RAP,” and a true target absorbed dose of [^211^At]MABG [8]

First, we plotted values converted to logarithms of [^131^I]MIBG [8] corrected by the physical half-life of ^131^I and fitted by the linear function, which was “a t + b” (Fig. 6a). The fitted function was − 0.1006 *t* + 4.7768 (*R*^2^ = 0.926). From this relation, we estimated that the HL of the biological clearance of a reference, *T*_*bA*2_, was 6.89 h. Next, we drew the behavior map of the optimal timing on A_1_ labeled with ^211^At and with *T*_*bA*2_ of 6.9 h (Fig. 6b). From the behavior map, we decided that 5 h of optimal timing, assuming the HL of the biological clearance of a target, *T*_*bA*1_, was close to that of *T*_*bA*2._ We calculated the inverse of the RAP coefficient from the values of %ID/g in simulation datasets of radiolabeled compound A_1_ and radiolabeled compound A_2_, which was 0.76. Finally, we estimated an absorbed dose of 3.3 Gy of [^211^At]MABG [8], by multiplying the RAP coefficient by that of the physical HL-corrected absorbed dose, 2.7 Gy, which is a numerical integration of the physical part of Eq. (5) (Fig. 6c). The difference between the RAP-converted absorbed dose and a true [^211^At]MABG [8] absorbed dose was 7%, which was superior to the 10% prediction error obtained by the representative RAP coefficient in Table 1. This result suggests that the present RAP dose conversion method is practical enough to use.

## Discussion

In this study, we focused on the mathematical ambiguities we encountered in our derivation of RAP coefficients in our previous work. We aimed to describe the RAP method mathematically without proportional relations and to examine the timing of a single biodistribution measurement (%ID/g). Here, we proposed a novel formula to estimate the optimal timing of a single measurement of %ID/g. Behavior analysis revealed the relation on the the optimal timing between the physical HL time of a target radiolabeled compound and the biological clearances of both the target and reference radiolabeled compounds. Finally, we presented an example of a practical use of RAP dose conversion.

A behavior map of an optimal timing on a single measurement of %ID/g might lead to several important strategies for RAP dose conversion. The first strategy involves a selection of a long biological clearance of a reference against a target with short and long physical HLs. If we could select a long biological clearance of a reference, we would have a broad window for optimal timing of a single biodistribution measurement (Figs. 3 and 4). For example, in the case of a target with 10 d physical HL, over 25 h of the biological clearance HLs of a target and a reference could make an almost 10-h window with 10% prediction error for optimal timing (Fig. 5a). The second is the operation of the short biological clearance HLs of a target and of a reference as shown in Fig. 5b. Even with a long physical HL, e.g., ^225^Ac, it might be possible to obtain the RAP coefficient within a feasible and short time by this operation, although it would be limited to a case of appropriate drug delivery. For example, the HLs of the biological clearance of nuclear medicine using the prostate-specific membrane antigen (PSMA) in animal studies were around 1 h [13]. A behavior map of an optimal timing might make it possible to present an appropriate imaging plan with a single measurement biodistribution for nuclear medicines that are difficult to measure multiple times.

We should note that RAP dose conversion using a behavior map did not require difficult formulas and techniques. We would need only the physical HL correction, linear fitting, the calculations of Eq. (8), and a numerical integration such as a trapezoidal quadrature. For example, most researchers would use absorbed dose estimation software, e.g., Organ Level INternal Dose Assessment/EXponential Modeling (OLINDA/EXM) (Vanderbilt University, Nashville, TN, USA) [14]. Using these programs, users estimate the time activity curve (TAC) of a radionuclide-labeled compound fitted by the exponential functions. The value per unit weight of the TAC corresponds to *C(t)* in the present Eq. (4) and input for the example as shown in Fig. 6, indicating the feasibility of RAP dose conversion. OLINDA/EXM users could also use RAP dose conversion.

We also should note that the optimal timing depended on the biological clearance, *T*_*bA*2_. The optimal timing of 4 h for the present simulation datasets was enough for 8 organs and tissue. However, if the value varies greatly from organ to organ, we need to adopt optimal timing values specific to organs of interest.

Unfortunately, clinical RAP coefficient information does not exist, and consideration of the RAP coefficient in animal biodistribution studies is currently limited. Also, an approximation of the one-compartment model applied to a behavior map of an optimal timing might create limitations in use. However, even in the biological clearance of [^131^I]MIBG [8] with a two-compartment phase [15], the RAP dose conversion displayed superior prediction error of less than 10%. The approach presented in a behavior map of an optimal timing on a single biodistribution measurement might provide useful information for the treatment planning of ^211^At-MABG therapy or TAT. Taken together, these results underscore the importance of developing the RAP dose conversion method for nonclinical and clinical future studies.

## Conclusions

The RAP dose conversion method renovated by the new formalism was able to estimate the [^211^At]MABG absorbed dose using the pharmacokinetics of [^131^I]MIBG through the use of a behavior map of an optimal timing of a single biodistribution measurement. The present formalism revealed optimizing imaging time points on absorbed dose conversion between two radiopharmaceuticals. Further analysis and clinical data will be needed to elucidate the validity of a behavior map of an optimal timing of a single measurement for TAT.

**Table 1:** Percent differences between converted absorbed doses and [^211^At]MABG [8] absorbed dose

Organ or tissue	Converted by	[^131^I]MIBG [8]	[^211^At]MABG [9]	[^125^I]MBBG [10]	[^77^Br]MBBG [10]
Heart	_HL_	− 19	21	− 11	3
	_HL+_ _RAP_	10	− 7	− 5	− 5
Liver	_HL_	− 21	4	− 29	− 30
	_HL+_ _RAP_	10	− 10	1	1
Kidney	_HL_	− 29	− 14	− 53	− 53
	_HL+_ _RAP_	10	− 9	1	− 1
Intestine	_HL_	− 6	19	− 20	− 22
	_HL+_ _RAP_	3	− 16	− 9	− 8
Blood	_HL_	− 36	13	2	5
	_HL+_ _RAP_	12	− 2	2	− 2
Stomach	_HL_	− 4	69	20	65
	_HL+_ _RAP_	− 1	− 1	0	0
Adrenals	_HL_	− 57	− 16	− 51	− 65
	_HL+_ _RAP_	10	− 8	− 2	− 2
Tumor	_HL_	− 17	797	569	694
	_HL+_ _RAP_	− 1	0	− 1	− 1
Average	_HL_	− 23	112	53	74
	_HL+_ _RAP_	7	− 7	− 2	− 2

## Supplementary Information

**Additional file 1**. Supplemental methods.

## Data Availability

The datasets used and/or analyzed in the present study are available from the corresponding author on reasonable request.
