# Supplementary material for: Nonclinical study and applicability of the absorbed dose conversion method with a single biodistribution measurement for targeted alpha-nuclide therapy
Source: EJNMMI Phys. 2021 Dec 11;8:80. doi: 10.1186/s40658-021-00425-z (PMC8665908; doi:10.1186/s40658-021-00425-z)
Supplement: Supplementary file 1 — Additional file 1. Supplemental methods. [file 40658_2021_425_MOESM1_ESM.pdf]

Supplemental methods

Optimal timing analysis method

Assuming the one-compartment model for biological clearance, the percent injected dose per gram $\%\mathrm{ID/g}$ of radiolabeled compounds A_1 and A_2 is expressed by the following respective equations:

$$(\%\mathrm{ID/g})_{A_1}(t) = \frac{A_0\_A_1 exp(-\frac{ln(2)}{T_{bA1}}t)}{IAC_0\_A_1}, \quad (s1)$$

$$(\%\mathrm{ID/g})_{A_2}(t) = \frac{A_0\_A_2 exp(-\frac{ln(2)}{T_{bA2}}t)}{IAC_0\_A_2}, \quad (s2)$$

where $A_0\_A_1$ and $A_0\_A_2$ are the initial activity concentrations, and T_{bA1} and T_{bA2} are HLs for the biological clearances of A_1 and A_2, respectively. Then, the activity concentration of A_1, $C\_A_1(t)$, and that of A_2 with HL, $C\_A_2\, with\, HL\ (t)$ are expressed by the following respective equations:

$$C\_A_1(t) = A_0\_A_1 exp(-\frac{ln(2)}{T_{pA1}}t)\ \ exp(-\frac{ln(2)}{T_{bA1}}t), \quad (s3)$$

$$C\_A_2\, with\, HL\ (t) = A_0\_A_2 exp(-\frac{ln(2)}{T_{pA1}}t)\ \ exp(-\frac{ln(2)}{T_{bA2}}t). \quad (s4)$$

The TIAC of A_1, $TIAC\_A_1$, and that of A_2 with HL, $TIAC\_A_2\, with\, HL$, were calculated by integrating equation (s3) and equation (s4), respectively:

$$TIAC\_A_1 = \int_{t_0}^{t_1} C\_A_1(t)\ dt\ = \frac{A_0\_A_1}{-\ln 2\left(\frac{1}{T_{pA_1}}+\frac{1}{T_{bA_1}}\right)}\left[\exp\left(-\ln 2\left(\frac{1}{T_{pA_1}}+\frac{1}{T_{bA_1}}\right)t\right)\right]_{t_0}^{t_1}, \quad (s5)$$

$$TIAC\_A_2 with\ HL = \int_{t_0}^{t_1} C\_A_2\ with\ HL\ (t)\ dt$$

$$= \frac{A_0\_A_2}{-\ln 2\left(\frac{1}{T_{pA_1}}+\frac{1}{T_{bA_2}}\right)}\left[\exp\left(-\ln 2\left(\frac{1}{T_{pA_1}}+\frac{1}{T_{bA_2}}\right)t\right)\right]_{t_0}^{t_1}, \quad (s6)$$

where t_0 and t_1 are the start and end times of the evaluation period.

Next, the RAP coefficient at time t is expressed by the following equation, assuming that $IAC_0\_A_1$ is equal to $IAC_0\_A_2$:

$$\text{RAP coefficient} = \frac{1}{\frac{(\%\text{ID/g})_{A_2}(t)}{(\%\text{ID/g})_{A_1}(t)}} = \frac{A_0\_A_1 exp(-\frac{ln(2)}{T_{bA1}}t)}{A_0\_A_2 exp(-\frac{ln(2)}{T_{bA2}}t)}. \quad (s7)$$

Finally, the optimal timing, *Opt_t*, was solved by inserting equations (s5) – (s7) into equation (8) and taking the natural logarithms of both sides.

Infinite end time of the evaluation period

The evaluation period for optimal timing equation (8) could have an infinite end time. If a one-compartment assumption for biological clearance holds for infinite time, the optimal timing equation could be transformed as follows:

$$Opt\_t = \frac{1}{-\ln 2\left(\frac{1}{T_{bA_1}}-\frac{1}{T_{bA_2}}\right)} \ln \frac{\frac{1}{T_{pA_1}}+\frac{1}{T_{bA_2}}}{\frac{1}{T_{pA_1}}+\frac{1}{T_{bA_1}}}, \quad (s8)$$

where *Opt_t* is an optimal timing, T_{pA1} is the physical HL of a target, T_{bA1} is the biological clearance HL of a target, and T_{bA2} is the biological clearance HL of a reference. However, verifiable data are rarely available for infinite time. In this paper, we proposed equation

(8) to calculate the optimal timing with a limited evaluation period, so that we could apply it even when the activity concentration data are available for only a short evaluation period. In fact, this was the case with our simulations of all datasets. In addition, an optimal timing formulated by equation (8) has limited application because of the assumption of the one-compartment model for biological clearance. This one-compartment assumption does not hold in the early stages of an evaluation period, when the uptake of radiolabeled compounds continues for a long time, as in equation (3). Therefore, in this case it is necessary to adjust the start time of the evaluation. This is an issue to be addressed in the future.
